# Ancient schwannoma of the cervical sympathetic chain

**DOI:** 10.1002/ccr3.971

**Published:** 2017-05-16

**Authors:** Philippe F. Bowles, Ryan Chin Taw Cheong, Samuel Cartwright, Andrew Pelser

**Affiliations:** ^1^ Colchester Hospital University NHS Foundation TrustTurner RoadColchesterEssexCO4 5JLUK; ^2^ Guy's and St Thomas’ NHS Foundation TrustGreat Maze PondLondonSE1 9RTUK; ^3^ Brighton & Sussex University Hospitals NHS Trust177 Preston RdBrightonBN1 6AGUK

**Keywords:** Ancient, cervical sympathetic chain, neurilemmoma, schwannoma

## Abstract

Ancient schwannomas exhibit characteristic histological features. Fine‐needle aspiration cytology (FNAC) is of limited use. Radiological evidence demonstrating displacement of blood vessels may aid diagnosis of schwannoma. Malignant transformation of ancient schwannoma has been reported. Surgical excision carries a high risk of postoperative Horner's syndrome.

## Introduction

Schwannomas are typically benign, slow‐growing, solitary neoplasms of Schwann cell origin. They were first described by Verocay in 1908 1 and have since been given several names although schwannoma and neurilemmoma are the most common terms. It is estimated that 25–40% of schwannomas occur in the head and neck 2 although schwannomas arising from the cervical sympathetic chain are uncommon 3. The term “ancient” schwannoma was introduced by Ackerman and Taylor in their description of 10 benign tumors of the thorax demonstrating extensive hyalinization suggestive of degenerative changes 4. The first case of an ancient schwannoma of the cervical sympathetic chain was described by Badawi in 2002 5.

## Case Presentation

A 36‐year‐old female, nonsmoker, presented to the head and neck rapid access clinic with a 1‐month history of a painless, left‐sided, neck lump. There was no preceding illness or features in the history suggestive of malignancy. Examination confirmed the presence of a soft, mobile left level II neck lump measuring approximately 2.5 cm in diameter. There were no overlying skin changes. Flexible nasendoscopy and examination or the oropharynx was normal.

**Figure 1:**
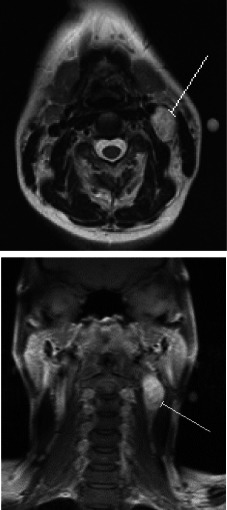
Axial and coronal views of contrast enhanced MRI (labels to ancient schwannoma).

Ultrasonography showed an isolated pathological left level II node measuring 1.4 cm in the short axis but no other pathological nodes. FNAC was nondiagnostic, producing hemorrhagic aspirate with inadequate cellular yield. Due to the proximity of the lymph node to the carotid sheath, magnetic resonance imaging (MRI) scan was chosen in preference to ultrasound‐guided Trucut biopsy (Fig. 1). This showed a 4.8 × 1.8 × 2.1 cm level II heterogenous soft tissue mass arising from the carotid space, abutting the carotid artery extending superiorly to the level of the hypopharynx and inferiorly to the infrahyoid region with a long cranial and caudal tail. A radiological diagnosis of schwannoma was made with histological diagnosis advised.

**Figure 2:**
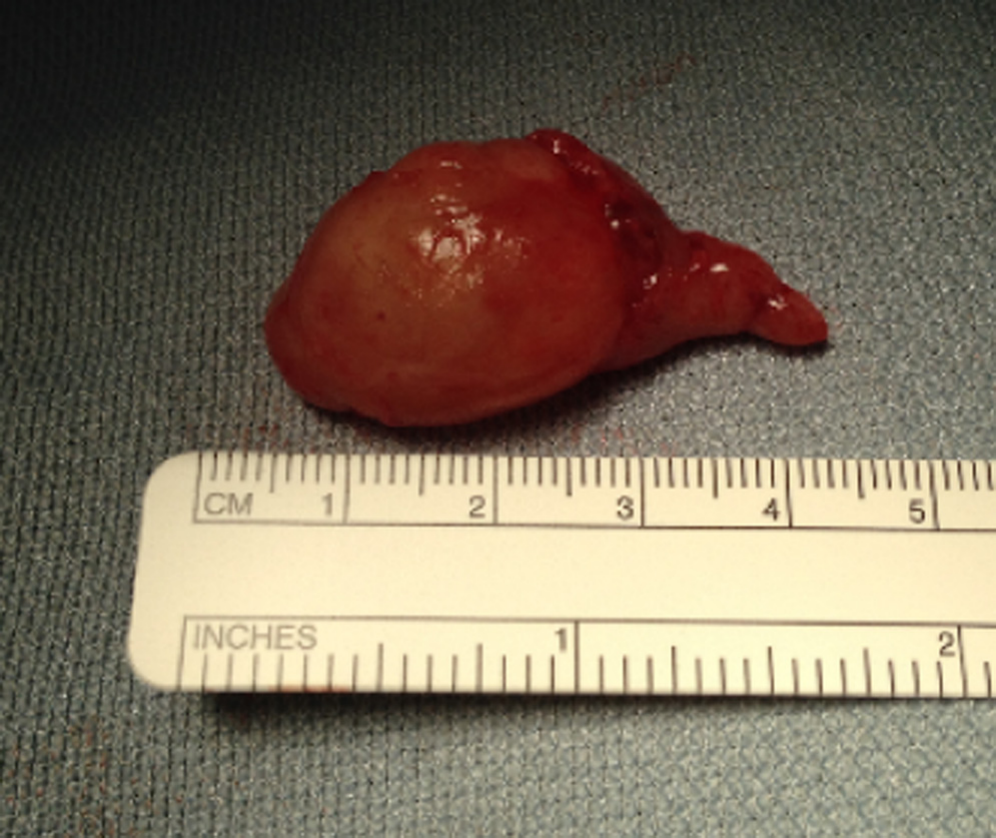
Excised mass prior to being sent for histology.

At surgery, the neck mass, arising from the cervical sympathetic chain, was accessed via a transverse neck incision over the anterior border of sternocleidomastoid muscle at an appropriately safe level parallel and 3 cm inferior to the mandible to protect the marginal mandibular nerve. The incision was made in an existing skin crease for optimal cosmetic outcome. The mass was carefully dissected from the nerve sheath minimizing trauma to the nerve tissue and sent for histological diagnosis (Fig. 2). The histology report confirmed a diagnosis of ancient schwannoma with the presence of classical Verocay bodies and thick‐walled vessels. The postoperative course was smooth with no complications reported at 6‐week follow‐up, and the patient was subsequently discharged.

## Discussion

A PubMed and MEDLINE literature search was performed with keywords “ancient,” “schwannoma,” “neurilemomma,” and “cervical sympathetic chain.” Manual cross‐referencing was also performed; three cases of ancient schwannoma of the cervical sympathetic chain were identified (Table 1) 2, 5, 6.

**Table 1:** Reported cases of ancient schwannoma of the cervical sympathetic chain

Author	Patient	Signs/Symptoms	Investigations	Initial Diagnosis	Management	Outcome
Badawi et al. 2002	23‐year‐old female	Asymptomatic unilateral neck mass nonpulsatile, nontender mass at the root of the left anterior triangle no lymphadenopathy no neurological deficit	Thyroid function tests Autoantibody screens Fine‐needle aspiration cytology (FNAC) Tc99 m thyroid scan	Thyroid mass	Surgical excision via external approach	Postoperative Horner's syndrome
Athar et al. 2007	41‐year‐old female	20 years history of nontender right upper neck swelling no neurological deficit	Fine‐needle aspiration (FNA) Computerized Tomography (CT)	Parapharyngeal mass	Surgical excision via external approach	Postoperative Horner's syndrome
Bihani et al. 2015	50‐year‐old female	2 year history of nonpulsatile, nontender mass at the anterior border of the right sternocleidomastoid no neurological deficit	Fine‐needle aspiration cytology (FNAC) Computerized tomography (CT) Magnetic resonance imaging (MRI)	Carotid body tumor	Surgical excision via external approach	Postoperative Horner's syndrome

Ancient schwannomas may be confused with cystic neck masses due to the typical radiological findings showing degenerated areas and nondiagnostic hemorrhagic aspirate with FNAC. Common differential diagnoses include carotid body tumors, parapharyngeal masses, tubercular lymphadenopathy, and sarcomas.

Although tissue biopsy and histology are required to confirm the diagnosis of ancient schwannoma, radiological evidence showing displacement of the carotid artery and internal jugular vein by the lesion may aid diagnosis of schwannomas. In schwannomas of the vagus nerve, the schwannoma grows between the common or internal carotid artery and the internal jugular vein, resulting in an increase in the distance between the artery and vein. In schwannomas of the cervical sympathetic chain, no separation is observed between the internal jugular vein and the common or internal carotid artery 7. Splaying of the carotid bifurcation (“Lyre” sign) with hypervascularity is usually due to a carotid body tumor 8.

**Figure 3:**
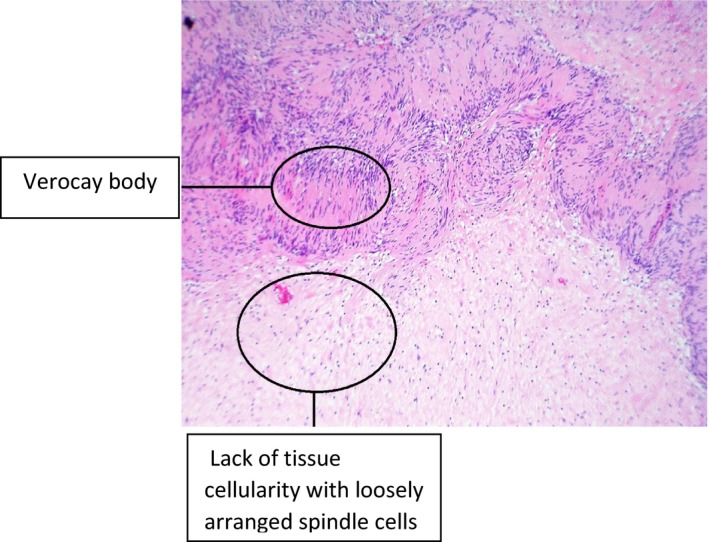
Histological features of ancient schwannoma (predominantly type B Antoni).

There are two histological types of schwannoma. Type A Antoni and type B Antoni. Type A Antoni are characterized by centrifugally compacted arrangements of spindle cells and Verocay bodies while type B Antoni are characterized by the lack of tissue cellularity and myxoid with loosely arranged spindle cells 3. The histopathology in ancient schwannoma, as in the case we report, shows relative loss of Antoni A areas with irregular nuclei and areas of hyalinization with hyperchromatism suggestive of degenerative changes (Fig. 3). Aside from nuclear atypia, additional changes associated with the degenerative process include formation of cysts, stromal edema, xanthomatous change, and fibrosis 9.

Although the tumor's slow growth, low recurrence rate, and noninvasive nature mean that conservative management may be considered as a treatment option, malignant transformation has been reported in at least nine cases of schwannoma 10 and one case of ancient schwannoma 11. Schwannomas are radioresistant, and therefore, surgical excision via an external approach is the treatment of choice. The most common complication associated with surgical excision of schwannomas of the cervical sympathetic chain is total or partial Homer's syndrome (ptosis, miosis, and anhydrosis).

## Key Learning Points

1. Ancient schwannomas exhibit characteristic histological features, and tissue biopsy is required for formal diagnosis.
2. Fine‐needle aspiration cytology (FNAC) yields nondiagnostic hemorrhagic aspirate and is of limited use.
3. Radiological evidence of displacement of blood vessels can aid with the differential diagnoses of schwannomas
4. Malignant transformation in an ancient schwannoma has been reported.
5. Careful dissection of the mass from the nerve sheath is required to reduce the risk of postoperative Horner's Syndrome.

## Authorship

PFB: design, literature review, and quality assurance of manuscript. RCTC: design, literature review, write‐up and quality assurance of manuscript. SC: design, literature review and quality assurance of manuscript. AP: design, quality assurance and principal surgeon on clinical case.

## Conflict of Interest

The authors declare that there is no conflict of interest regarding the publication of this paper.
